# Ultrasound Elastography for the Evaluation of Lymph Nodes

**DOI:** 10.3389/fonc.2021.714660

**Published:** 2021-08-17

**Authors:** Bin Wang, Qi Guo, Jia-Yu Wang, Yang Yu, Ai-Jiao Yi, Xin-Wu Cui, Christoph F. Dietrich

**Affiliations:** ^1^Department of Ultrasound, The First People’s Hospital of Yueyang, Yueyang, China; ^2^Department of Medical Ultrasound, The Fifth Affiliated Hospital of Zhengzhou University, Zhengzhou, China; ^3^Department of Medical Ultrasound, Tongji Hospital, Tongji Medical College, Huazhong University of Science and Technology, Wuhan, China; ^4^Department of Internal Medicine, Hirslanden Clinic, Bern, Switzerland

**Keywords:** lymph nodes, screening, ultrasonography, elastography, shear wave

## Abstract

The differential diagnosis of lymphadenopathy is important for predicting prognosis, staging, and monitoring the treatment, especially for cancer patients. Conventional computed tomography and magnetic resonance imaging characterize lymph node (LN) with disappointing sensitivity and specificity. Conventional ultrasound with the advantage of high resolution has been widely used for the LN evaluation. Ultrasound elastography (UE) using color map or shear wave velocity can non-invasively demonstrate the stiffness and homogeneity of both the cortex and medulla of LNs and can detect early circumscribed malignant infiltration. There is a need of a review to comprehensively discuss the current knowledge of the applications of various UE techniques in the evaluation of LNs. In this review, we discussed the principles of strain elastography and shear wave-based elastography, and their advantages and limitations in the evaluation of LNs. In addition, we comprehensively introduced the applications of various UE techniques in the differential diagnosis of reactive LNs, lymphoma, metastatic LNs, and other lymphadenopathy. Moreover, the applications of endoscopic UE and endobronchial UE are also discussed, including their use for improving the positive rate of diagnosis of fine-needle aspiration biopsy.

## Introduction

Various benign and malignant disorders can result in lymphadenopathy; the differential diagnosis of lymph node (LN) is important for predicting prognosis, staging, and monitoring the treatment. Conventional computed tomography (CT) and magnetic resonance imaging (MRI) characterize LN relying on size and topographic distribution, but with disappointing sensitivity and specificity, since it is not rare that malignant LN infiltration occurs in normal-sized LN.

**Figure 1:**
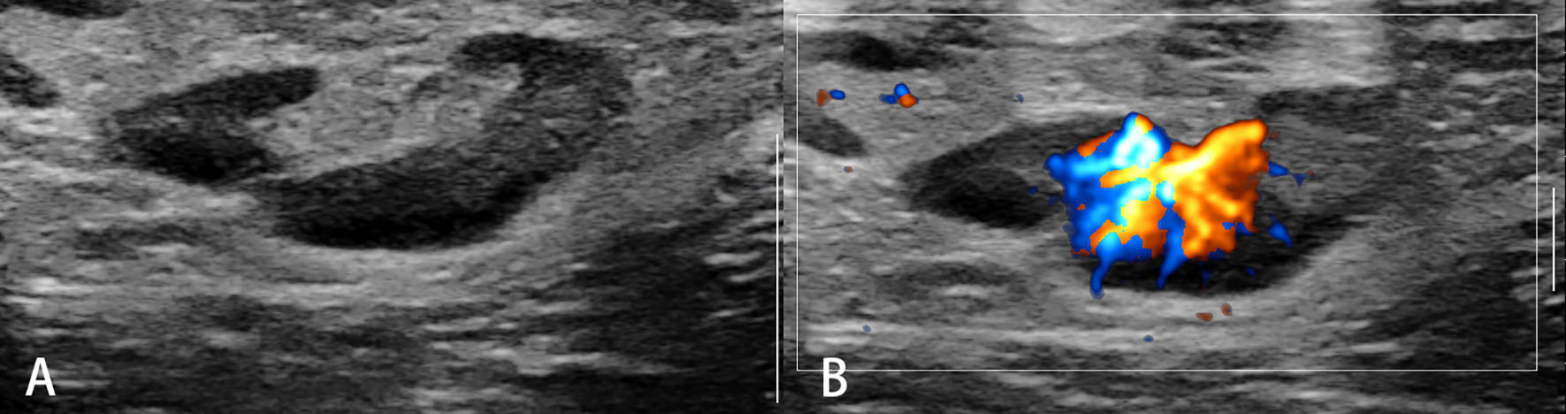
Reactive lymph nodes. Typically, the architecture **(A)** and predominant vessel architecture **(B)** are preserved.

**Figure 2:**
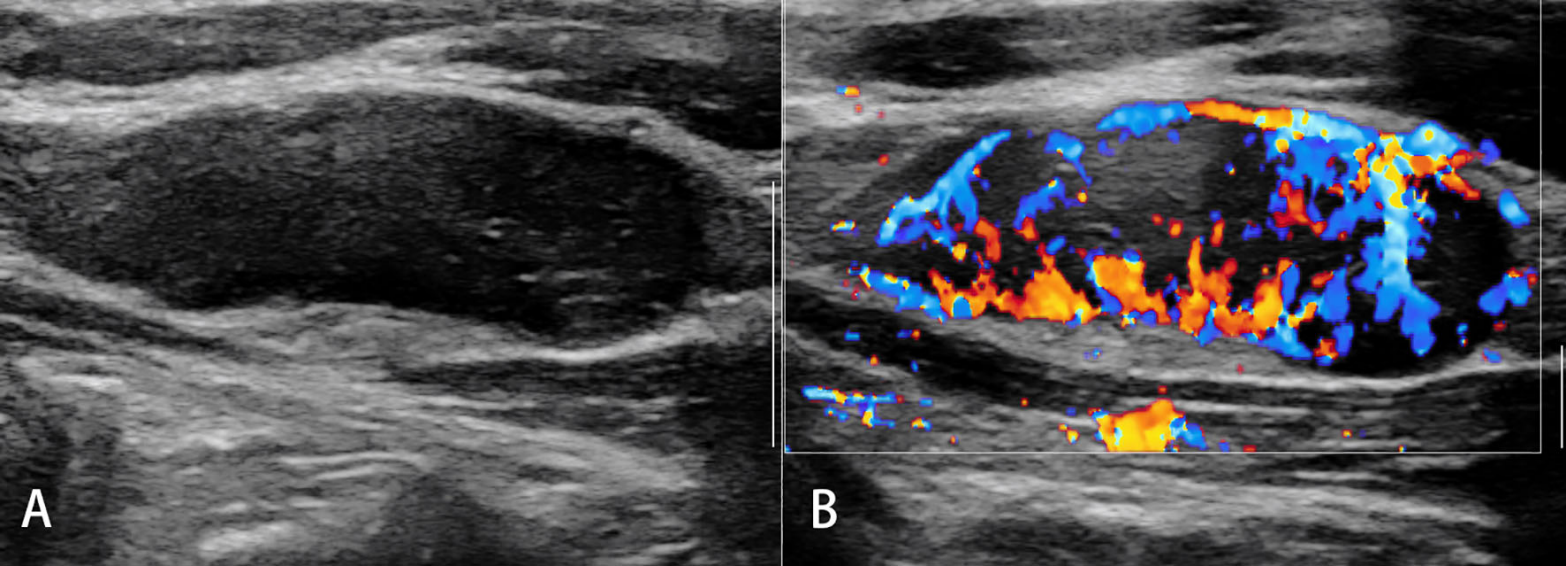
Malignant lymph nodes (carcinoma infiltration). The eccentric hypoechoic cortical thickening **(A)** and vessel destruction **(B)** in the lymph node are observed.

**Figure 3:**
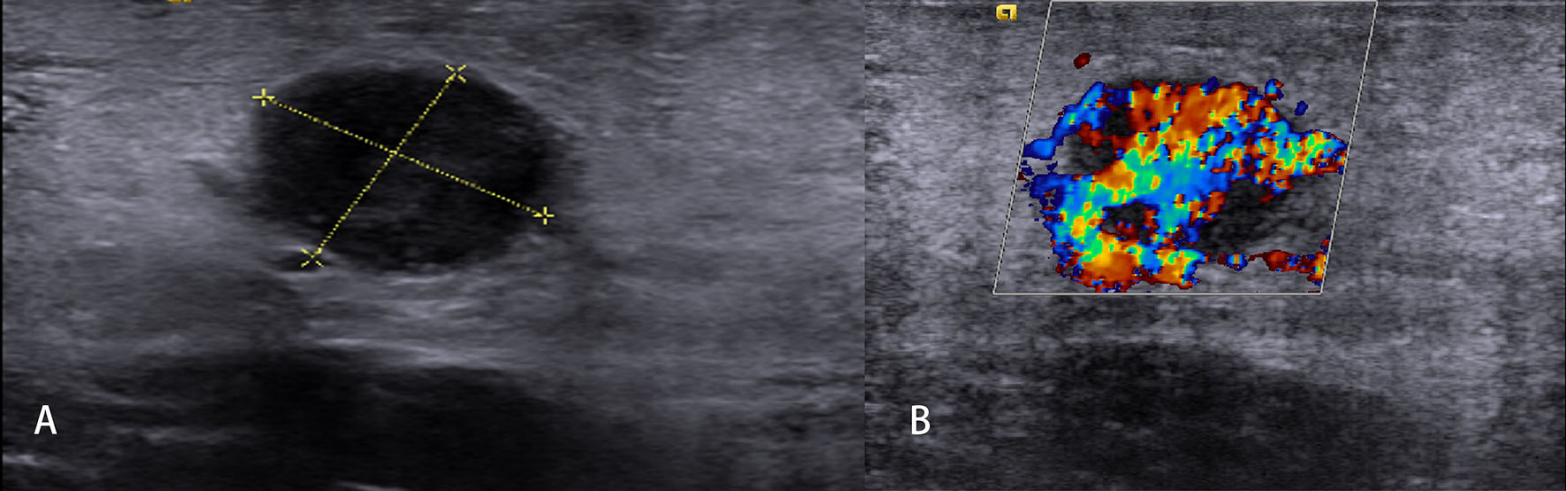
Non-Hodgkin’s lymphoma. The destroyed architecture, approximate sphere, and pseudocystic appearance **(A)** and rich vascularity **(B)** are observed.

Conventional ultrasound (US) with the advantage of high resolution has been widely used for imaging superficial organs, particularly for the LN evaluation. Compared with conventional CT and MRI, B-mode US can provide more detailed information on shape, contour, inner texture, maximum short axis diameter, long to short axis ratio, absence of hilus, and presence of necrosis. Color Doppler US and spectral Doppler US can image the hemodynamic characters of LN and add values for the differentiation of malignant from benign LNs. Benign LNs often show hilar predominant vessel architecture and have lower resistive index (RI), while malignant LNs usually show peripheral or mixed vascularity and disappearance of hilar vascularization and have higher RI. However, Doppler techniques have limitations in small LN since the vascularity is often undetectable (**Figures 1**–**3**).

US elastography (UE) is a new technique that uses color map or shear wave velocity (SWV) to non-invasively demonstrate stiffness and homogeneity. It has rapidly become one of the most popular US-based techniques. Clinically, it can be used in the early detection and differential diagnosis of focal diseases; in improving the accuracy for diagnosing diffuse diseases, such as fibrosis and atherosclerosis; and in the assessment of response to treatments, such as thermal ablation and chemotherapy (1).

UE is able to demonstrate the stiffness of both the cortex and medulla of LNs and to detect early circumscribed malignant infiltration. Studies have been published on the evaluation of LNs by strain elastography (SE) or shear wave elastography (SWE). This review aims to comprehensively discuss the current knowledge of the applications of various UE techniques in the evaluation of LNs.

## Principles and Techniques of Ultrasound Elastography

UE is a technique in which the stiffness of the tissue can be imaged as color map or SWV. The principle of UE is based upon tissue reactions, such as changes in displacement, strain, or speed, by applying an external or internal static (quasi-static) or dynamic excitation. Differences in tissue reactions are calculated, identified, and reflected by computers.

**Figure 4:**
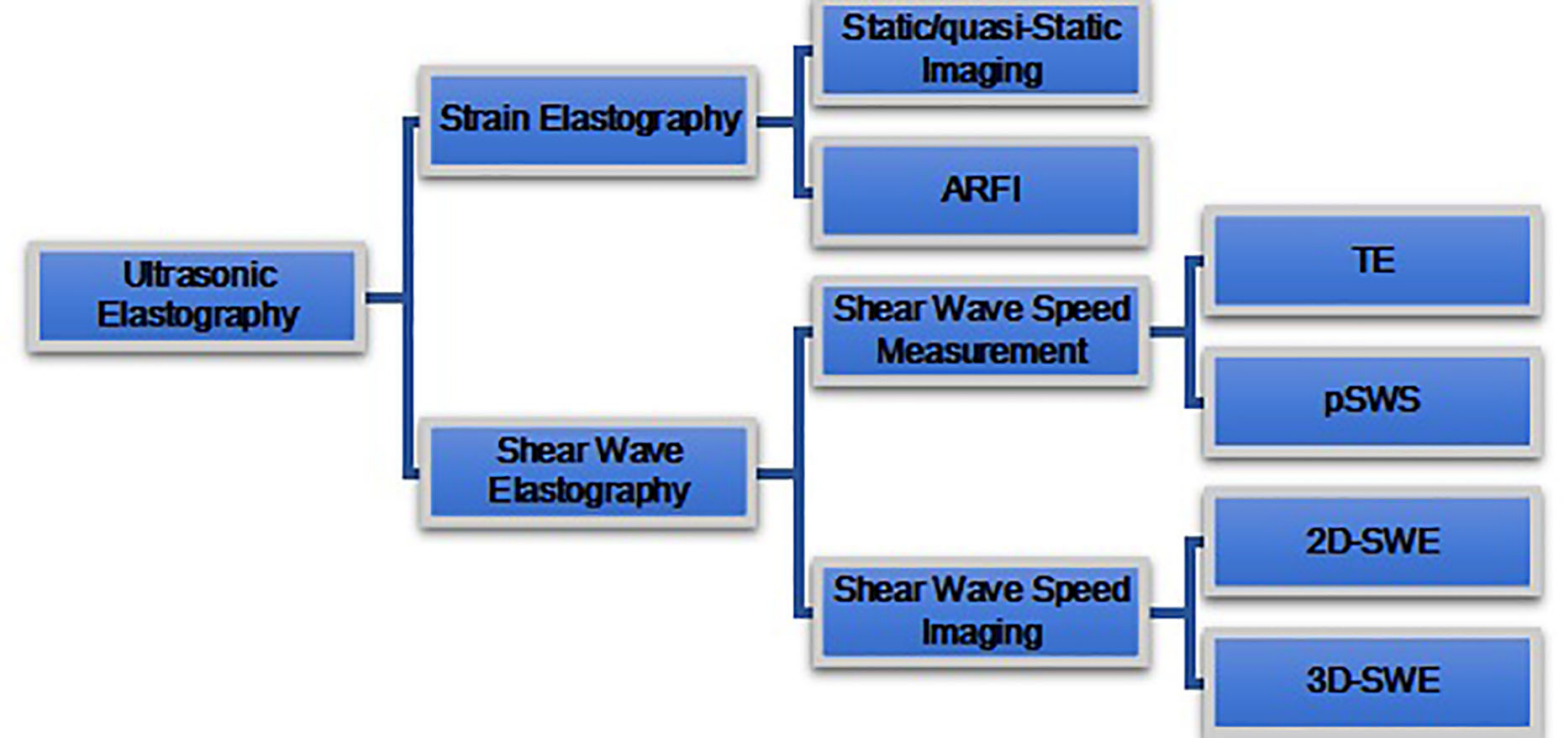
Ultrasound elastography (UE) techniques. UE techniques can be classified by the type of excitation applied: 1) strain elastography (top) and 2) shear wave elastography (bottom). Excitation methods of strain elastography include constant force-induced displacement (static/quasi-static imaging) or acoustic energy-induced physiologic motion (ARFI). Excitation method of shear wave elastography where the shear waves are produced by a transducer. Shear wave elastography is classified as transient elastography (TE), point shear wave elastography (pSWE), two-dimensional shear wave elastography (2D-SWE), and three-dimensional shear wave elastography (3D-SWE), according to different measurement and imaging methods.

Depending on the type of excitation applied, UE is classified into two categories, i.e., 1) SE, which is composed of static or quasi-static strain imaging and acoustic radiation force impulse (ARFI) imaging; and 2), SWE which is composed of SWV measurement and SWV imaging (**Figure 4**).

### Strain Elastography

#### Technique

SE includes static/quasi-static imaging and ARFI imaging. It is based upon the fact that hard tissue is more difficultly compressed than soft tissue. SE is a technique that measures the tissue deformation generated by compression, which may be applied with a probe on the body surface for static/quasi-static imaging and may also be applied with acoustic radiation force for ARFI imaging. The tissue deformation is measured by US system and displayed as a color or gray map. On the screen of the US system, both the B-mode image and corresponding elastography image could be simultaneously displayed.

The parameters commonly used to indicate tissue hardness include elasticity score and strain ratio (SR). The elasticity score indicates the strain (with color or brightness) distribution within a selected area. The SR refers to the ratio of strain between area A (usually a mass) and area B (usually a normal surrounding tissue, fat, or muscle tissue) within the region of interest (ROI).

#### Advantages and Limitations

SE, especially static/quasi-static imaging, is suitable for superficial organs and thus is the most commonly used method for the evaluation of superficial LNs. The operation method of SE is simple, and the operation skills can be mastered in a short time of training. However, SE is a qualitative analysis technique, and it is not able to analyze tissue hardness quantitatively. The performance of static/quasi-static imaging is not good at analyzing the deep LNs. Moreover, SE is user dependent and subjective (2, 3).

### Shear Wave Speed Measurement

#### Techniques

Shear wave speed measurement technology is a method to generate shear waves and measure SWV. Based on the principle of fast propagation of shear wave speed in hard tissue and slow propagation in soft tissue, the hardness of tissue is indirectly reflected by measuring shear wave speed. Shear wave speed can be converted to Young’s modulus by Young’s model formula:

$$\text{E}=3\rho {\text{C}}^{2}$$

where E represents stiffness (Young’s modulus [kPa]), ρ is the density (kg/m^3^, approximately equal to 1), and C is the shear wave speed (m/s).

The shear wave speed measurement techniques mainly include transient elastography (TE) and point SWE (pSWE). TE is the first shear wave speed measurement technology applied in clinical practice, but it is only used in the liver so far; therefore, this technique is not discussed in this review. The principle of pSWE is similar with ARFI: the probe applies an acoustic radiation force to the ROI of the tissue and generates transverse vibration shear waves. The receiver can detect the speed of shear wave in ROI, which is expressed by speed or by kPa value through Young’s model formula.

#### Advantages and Limitations

pSWE can detect both deep organs (the liver, etc.) and superficial organs (the thyroid, etc.) (4–6), and therefore, this technique is suitable for both superficial and deep LNs. However, the ROI is with fixed size; it can only measure one part of a LN but may be too large if the LN is very small.

### Shear Wave Speed Imaging

#### Techniques

The principles of shear wave speed imaging are that the US probe sends out the multipoint focused acoustic radiation force pulse, which makes the tissues at different depths along with the acoustic axis shift at almost the same time, producing plane shear wave, and then the image processing technology detects the SWV, forms color image, and calculates Young’s modulus (elasticity index (EI)) (7). So compared with that of pSWE, the size of ROI in 2D-SWE can be adjustable. Some US diagnostic instruments are equipped with 3D probes with high-speed acquisition capability of mechanical scanning 2D-sensor sequences, which can conduct 3D reconstruction of tissue hardness.

#### Advantages and Limitations

The diagnosis of shear wave speed imaging is less influenced by the operator’s experience and operation than SE, because it does not rely on freehand compression (8). It can display the conventional US images and elastic US images synchronously and measure SWV in real time. However, multicenter studies have shown that the repeatability of shear wave elastic imaging is affected by the size, location, depth, and other factors (9, 10).

## Clinical Applications of Elastography

### Reactive Lymph Nodes (Inflammation)

**Figure 5:**
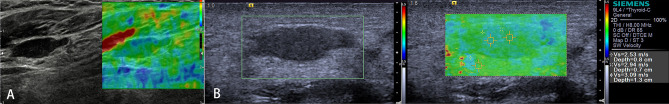
Reactive lymph nodes. Both the strain elastography **(A)** and shear wave-based elastography **(B)** reveal uniform and symmetrical soft tissue (green).

Acute or chronic inflammation is the prime cause of LN enlargement (**Figure 5**). The elastographic architecture of LNs is kept in most inflammatory processes. Therefore, like in normal LNs, the cortex is also stiffer than the hilum in inflammatory LNs.

#### Strain Elastography

Both elasticity score and SR have been studied to evaluate the stiffness of reactive LN. Firstly, due to the lack of a unified classification method for US elastograms, different researchers classified US elastograms of LNs into a 4-point, 5-point, 6-point, 7-point, or 8-point rating scale. Secondly, some researchers compared the strain in target region with adjacent reference region to differentiate benign from malignant LNs.

Lyshchik classified US elastograms of LNs with a 4-point rating scale according to visibility, brightness compared with surrounding neck muscles, regularity, and definition of outline (11). Several studies classified elastograms of the LNs into five patterns according to relative distribution and ratio of soft or hard regions of the LN: pattern 1, absent or very small blue (hard) areas; pattern 2, total blue areas of less than 45%; pattern 3, total blue areas of greater than 45%; pattern 4, peripheral blue area and central green (soft) area; and pattern 5, blue area with or without a green rim. Tan et al. found that 87.9% of benign LNs manifest pattern 1 or pattern 2 (3).

Besides, Lyshchik defined the surrounding neck muscles to LN SR as strain index; using strain index value of <1.5 in benign LN classification, SE showed 79% accuracy, 85% sensitivity, and 98% specificity (11). Acu et al. calculated each LN with mean strain index. With the use of strain index value of <1.7, SE differentiates benign LNs from malignant ones with 75% accuracy, 71.6% sensitivity, and 76.5% specificity (12). Özel et al. reported that elastography SRs were lower in benign LNs than malignant LNs (13). Many studies have shown that SE has potential diagnostic value in lymphadenopathy (**Table 1**); however, high user dependence is the limitation, especially using SR. Adjacent reference region was selected differently for SR measurement of LNs in different regions; in general, muscles as adjacent reference tissues were usable in cervical region, and fat tissue as an adjacent reference region may be a good choice in the axilla.

**Table 1:** The diagnostic performance of ultrasound elastography in differentiating benign and malignant LNs.

Study	Study description	LN	SE (%)	SP (%)	PPV (%)	NPV (%)	Accuracy	Gold standard
Lo WC, *European Radiology* (14)	Qualitative (4 patterns)	131	66.7	57.1	52.2	71.0	–	Histology
Suzan Onol, *Cureus* (15)	Qualitative (4 scores)	70	94	70	–	–	86	Histology
Leyla Acu, *J Ultrasound Med* (12)	Qualitative (5 patterns)	220	82.1	56.2	45.1	87.8	64.1	Histopathology
Tsai WC, *Ultrasound in Medicine & Biology* (16)	Qualitative (5 patterns)	90	86	90	91	84	88	Surgical pathology
Xu Y, *Scientific Reports* (17)	Qualitative (5 patterns)	97	78	93	93	79	86	Surgical pathology
Müberra Pehlivan, *Braz J Otorhinolaryngol* (18)	Qualitative (5 patterns)	16	82.4	84.6	87	78	83.3	Histology
Zhang F, *Medicine (Baltimore)* (19)	Qualitative (6 patterns)	97	81.58	95.65	–	–	86.89	Histology
Lenghel LM, *Medical Ultrasonography* (20)	Qualitative (8 patterns)	69	66.7	96.7	–	–	84.6	Follow-up, histology
Lyshchik A, *Radiology* (11)	Quantitative (strain index ≥ 1.5)	141	85	98	–	–	92	Histology
Leyla Acu, *J Ultrasound Med* (12)	Quantitative (strain index ≥ 1.7)	220	71.6	76.5	57.1	86.0	75.0	Histopathology
Müberra Pehlivan, *Braz J Otorhinolaryngol* (18)	Quantitative (strain index ≥ 1.04)	16	100	84.62	–	–	95	Histology
Zhang F, *Medicine (Baltimore)* (19)	Quantitative (SWV ≥ 2.76 m)	97	57.89	86.96	–	–	68.85	Histology
Fujiwara T, *Ultrasound in Medicine & Biology* (21)	Quantitative (SWV ≥ 1.9 m/s)	42	95.0	81.8	–	–	88.0	Surgical pathology, Lymph node open biopsy
Meng W, *European Journal of Radiology* (22)	Quantitative (VTIQ value ≥ 2.595 m/s)	181	82.9	93.1	–	–	90.6	Surgical pathology, fine-needle aspiration
Azizi G, *Ultrasound in Medicine & Biology* (23)	Quantitative (VTIQ value ≥ 2.93 m/s)	270	92.59	75.49	48.54	97.60	78.9	Surgical pathology
Zuhal Bayramoglu, *Br J Radiol* (24)	Quantitative (elasticity > 17 kPa) (lymphoma *vs*. lymphadenitis)	117	96	100	100	99	99	Histology
Shuyi Luo, *Front Oncol* (25)	Qualitative SWE (4 scores)	121	96.7	100	100	96.8	98.3	Core needle biopsy, surgical pathology
Wei Lin Ng, *Acad Radiol* (26)	Qualitative SWE (4 scores)	107	96.0	56.1	–	–	81.3	Histopathology

LNs, lymph nodes; SE, sensitivity; SP, specificity; PPV, positive predictive value; NPV, negative predictive value; SWV, shear wave velocity; VTIQ, virtual touch tissue imaging quantification; SWE, shear wave elastography.

#### Shear Wave-Based Elastography

Compared with SE, shear wave-based elastography is regarded as potentially more objective. In most published researches, virtual touch tissue imaging (VTI) grade and SWV of ARFI imaging were used to evaluate reactive LNs, and the diagnostic performance of VTI is higher than that of SWV (19). In a study including 263 pediatric LNs, Bayramoglu et al. found that median elasticity and velocity values were higher in reactive LNs compared with normal LNs; with the use of the cutoff median elasticity and velocity values of >15 kPa and 2.24 m sn^−1^ for differentiating reactive LNs from normal LNs, sensitivity, specificity, positive predictive value (PPV), negative predictive value (NPV), and diagnostic accuracy were 27%, 96%, 82%, 74%, and 74% and 25%, 97%, 82%, 73%, and 74%, respectively. Many studies should be conducted on the evaluation of reactive LNs by shear wave-based elastography to explore the significance of SWE in the evaluation of reactive LNs and to analyze the potential factors affecting SWE imaging (24).

### Malignant Lymph Nodes (Carcinoma)

**Figure 6:**
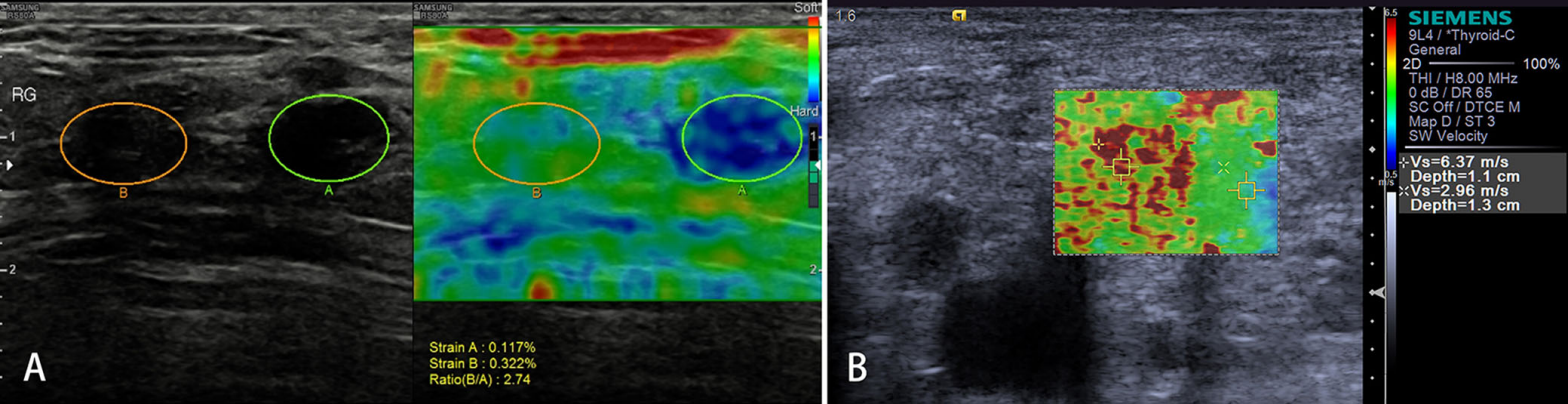
Malignant lymph nodes (LNs) (carcinoma infiltration). The strain elastography reveals typically harder (blue) area in the LN than the surrounding tissues (green); strain ratio = 2.74 **(A)**. The shear wave-based virtual touch tissue imaging quantification reveals a harder (red) area in the LN, and the maximum shear wave velocity (6.37 m/s) is much higher than that of surrounding tissues (2.96 m/s) **(B)**.

Malignant tumor cells proliferate rapidly, causing internal pressure and increasing tissue stiffness in LNs. Therefore, the elastographic architecture of LNs changed compared with reactive LNs. Typically, the well-differentiated carcinoma initially infiltrates LNs in a circumscribed manner (focally stiffer and harder), whereas the undifferentiated carcinoma leads to a diffuse (mostly or completely stiffer and harder) infiltration (**Figure 6**).

#### Strain Elastography

Several pilot studies have evaluated the ability of SE to detect LN metastases in the cervical or axillary LNs (11, 14, 20–22, 27). Both elasticity score and SR have been studied, which showed that SE and conventional US may play complementary roles in differentiating malignant LNs and assessing the risk of metastatic LNs.

Firstly, suspected cervical LN metastases from hypo-pharyngeal and thyroid carcinomas have been recently investigated using SE (real-time elastography (RTE)) (11). An EI has been created by comparing the elasticity of the LN with the surrounding head and neck muscle tissue (muscle to LN SR). With the use of a ratio of >1.5 as an indicator of malignant infiltration, the sensitivity was 85% and the specificity was 98%, which are superior to the best B-mode criteria (11). These data have been reproduced by Tan et al. Moreover, inter-observer agreement with SE was very high (kappa 0.88–0.946) (3).

Secondly, some researchers qualitatively classified US elastograms of LNs into a 4-point, 5-point, 6-point, 7-point, or 8-point rating scale. Metastatic LNs were mostly evaluated to 3–4 points in a 4-point rating scale. Suzan (15) found that the sensitivity, specificity, and accuracy of RTE in differentiating benign LN from squamous cell carcinoma and malignant melanoma group were 91%, 70%, and 86%, respectively.

In a 5-point rating scale (3, 12, 16, 18), Tan et al. reported that 50 of malignant and 74.5% of metastatic LNs manifested pattern 3 or 4, while all primary malignant LNs manifested pattern 2 (3). In another study including 97 axillary LNs, using the criteria of score 1 and 2 as benign and scores 3, 4, and 5 as metastatic, the sensitivity, specificity, PPV, NPV, and accuracy were 78%, 93%, 93%, 79%, and 86%, respectively (16). Although qualitative strain methods based on elasticity score and SR have been widely studied all over the world for axillary and cervical LNs (17), SR >1.5 or hard composition over 50% can be a good indicator of malignancy. However, as compared with SWE, its dependence on operators cannot be overcome, and absolute quantitative elastic measurement cannot be provided; and for LNs with deep vertical distance and small volume, the judgment of RTE on LNs hardness is prone to false-positive results, which affects the accuracy of SE (16).

#### Shear Wave-Based Elastography

Clinically and theoretically, SWE seems to be an effective, quantitative tool for differential diagnosis of malignant and benign LNs in many researches, especially in small LNs (28). Based on previous researches, the maximum SWV (2.93 m/s) (23) and elastic value ratio (29) can be used as reliable indices to predict benign and malignant lymphatic nodes. Kılıç A et al. conducted a prospective study comparing conventional US with VTI quantification (VTIQ), and when using a cutoff value of 3.03 m/s, VTIQ differentiates malignant LNs from benign ones with 75% accuracy, 93% sensitivity, and 59% specificity (30).

Some researchers (25, 26) qualitatively classified SWE images of axillary LN (ALN) into 4-point patterns, which was similar to SWE patterns of breast lesions (31): color pattern 1, homogeneous pattern; color pattern 2, filling defect within LN; color pattern 3, homogeneous within LN with a localized colored area at the margin; and color pattern 4, filling defect within LN with a localized colored area at the margin (25). The benign ALNs usually manifest color pattern 1, while ALN metastases (ALNMs) usually manifest color patterns 2–4, and the sensitivity, specificity, PPV, NPV, and area under the receiver operating characteristic (ROC) curve (AUC) were 96.7%, 100%, 100%, 96.8%, and 98.3%, respectively (25). In addition, Luo et al. (25) and Lin et al. (26) directly compared the diagnostic performance of qualitative and quantitative SWE, and they found that qualitative SWE had better diagnostic performance than quantitative SWE in detecting ALNM.

However, a meta-analysis compared the diagnostic performance of qualitative elastography with quantitative elastography for ALNM in breast cancer and found that quantitative and qualitative elastography had similar diagnostic performance and good clinical utility (32). More studies with SWE should be conducted to get more reliable cutoff values of SWV and elastic value ratio in different sites.

### Lymphoma

**Figure 7:**
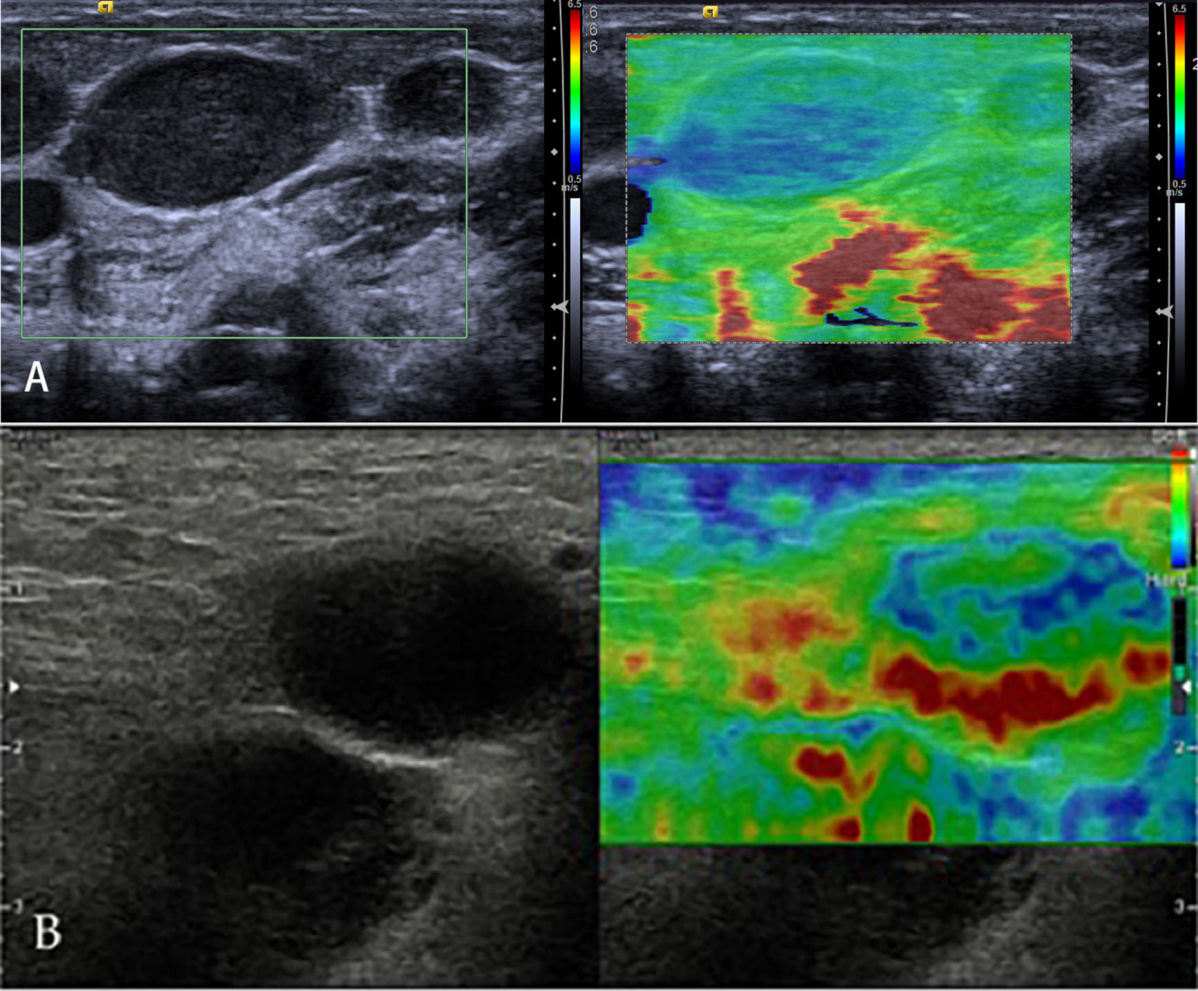
Non-Hodgkin’s lymphoma. The strain elastography reveals a focal harder (blue) area in the lymph node **(A)** and a diffuse harder (blue) area in the lymph node **(B)**.

Lymphomas are a primary malignant tumor of LNs, lymphoid tissues outside LNs, and mononuclear macrophage system (**Figure 7**). Because of a highly heterogeneous group of lympho-proliferative malignancies, the biological behavior and pathological types of lymphomas are different, especially for non-Hodgkin’s lymphoma. But the incidence of lymphomas represents approximately 4%, and newly diagnosed cancers increases each year; moreover, lymphomas are more commonly seen in developed countries, which may seriously endanger people’s health (33). Knowledge of elastography in lymphoma is very limited. So far, different lymphomas cannot be differentiated. Initial experience suggests that focal LN infiltration (**Figure 7A**) is indicative of low-grade follicular lymphoma, whereas diffuse and homogenous LN infiltration is typically found in high-grade lymphoma (**Figure 7B**).

#### Strain Elastography

Few studies have reported on the evaluation of lymphoma with SE. With a 5-point rating scale of US elastograms of LNs, Acu et al. reported that most lymphoma manifested patterns 1 and 2 (16). Clinically and theoretically, the stiffness degree of lymphoma is different from that of metastatic and benign LNs (34). In most studies, the hardness of the lymphoma was low. Thus, when metastasis and lymphoma were considered as positive, reactive LNs were considered as negative in the differential diagnosis; and the sensitivity, specificity, and accuracy with a point rating scale of US elastograms were affected (35). With quantitative analysis of SE, elasticity parameter strain index showed high diagnostic accuracy for distinguishing lymphoma from lymphadenitis; the cutoff value of the strain index of the cervical LNs compared with sternocleidomastoid muscle has been reported to be 1.18 in a recent study (36). Though it is difficult to differentiate different lymphomas, the treatment effect evaluation with SE in Hodgkin’s disease may be useful.

In the study on the efficacy of refractory and recurrent Hodgkin’s disease, it has been shown that the hardness of some lymphoma nodules changes with the treatment effect. It indicates that SE could be reliable for therapy response monitoring of Hodgkin’s lymphoma (37).

#### Shear Wave-Based Elastography

Currently, there are few studies on the evaluation of lymphoma by SWE. The number of enrolled lymphoma in these studies was small, including several case reports. Soo et al. qualitatively categorized shear speed map in a of total five SWE patterns in cervical LNs: pattern 1, absent or very small red (stiff) area; pattern 2, small scattered red areas, which mean total red area less than 45%; pattern 3, large red area, equal or more than 45%; pattern 4, peripheral red area and central green (soft) area, suggesting central necrosis; and pattern 5, almost red area with or without a green rim. None of lymphoma manifested pattern 4 and pattern 5; and absolute values and ratio of both elasticity and speed were significantly lower in lymphomas than metastatic LNs (38). Based on a recent study in pediatric LN with quantitative evaluation of SWE, elasticity values higher than 17 kPa and velocity values higher than 2.45 m/s would be considered as lymphoma rather than lymphadenitis in an enlarged LN with at least a 91% diagnostic accuracy (24). Several case reports have evaluated uncommon different lymphomas with SWE. A report used SWE to evaluate primary B-cell lymphoma of the breast. The study showed that the mass of primary B-cell lymphoma on SWE was considerably stiff but softer than typical invasive ductal cancers. In the future, a prospective study with large-scale samples should be conducted to investigate quantitative or qualitative SWE features of primary B-cell lymphoma (39).

### Other Lymphadenopathy

In the preliminary study of reactive and metastatic LNs, the AUC for combined evaluation is 0.97, which is much higher than that for B-mode US or elastography alone (28). The analysis of parameters can be used to quantitatively evaluate the characteristics of different LN diseases; it shows that LNs of tuberculosis (TB) are softer than metastatic LNs but harder than benign LNs (40). However, LNs of TB have a wide range of stiffness; the stiffness is related with internal structures, increased fibrous tissue and calcification can account for high stiffness, and liquefaction necrosis can decrease the stiffness. Cheng et al. found that only 50% LNs of TB can be correctly diagnosed by elastography (41).

In further studies, the combination of B-mode US and elastography may have important clinical value in differential diagnosis. Few researches have been done on relapsing or chronic lymphadenitis or rare benign diseases such as Kikuchi or Kimura disease (KD). The research shows that the LNs with KD show malignant signs in conventional US, but benign signs in SE; therefore, SE can help patients avoid unnecessary needle biopsy and inappropriate treatment (42). In a study of children’s cervical LNs, the stiffness of the largest LNs in patients with bacterial cervical lymphadenitis (BCL) was significantly higher than that in patients with LN-first presentation of Kawasaki disease (NFKD) and healthy children, with a cutoff of 14.55 kPa; the sensitivity, specificity, and AUC were 89%, 76%, and 88.5%, respectively (43). So SWE is a potential method to differentiate early NFKD.

## Applications of Endoscopic Ultrasound and Endobronchial Ultrasound Elastography

Endoscopic US (EUS) and endobronchial US (EBUS) are important tools to assess the digestive tract and surrounding organs, but the limited capacity to determine the exact pathological results is the major limitation. As a non-invasive technique, EUS and EBUS elastography have been proven to be able to provide complementary stiff information added to conventional EUS and EBUS imaging, becoming promising examination methods to differentiate benign from malignant LNs (44–46).

### Differentiation of Benign and Malignant Lymph Nodes

Recently, an increasing number of literatures focused on the use of EUS and EBUS to diagnose mediastinal LNs and peritoneal lymphadenitis.

EUS elastography was originally used for the differential diagnosis of pancreatic lesions. Studies on the difference between benign and malignant pancreatic masses and LNs by SE showed that EUS elastography had more advantages than conventional US (47).

Similar to superficial LNs, physiological and reactive peritoneal LNs manifest homogeneous or scattered soft pattern with delineated vascular structures of LN hilum. And the LN medulla may manifest as slightly softer than the LN cortex. Malignant LNs are the most characterized by a homogeneous hard elastographic pattern, especially in diffuse metastatic infiltration; however, malignant LNs may display inhomogeneous but hard patterns because of incomplete metastatic infiltration and focal necrosis. More and more studies differentiated benign from malignant LNs with EUS; most of them were qualitative with elastographic histogram, using EUS–fine-needle aspiration biopsy (FNAB), histology, and/or surgical pathology as a reference standard.

Multiple studies have demonstrated that EUS and EBUS elastography can effectively identify the benign and malignant mediastinal and peritoneal LNs (**Table 2**) (47–51, 53–59). In addition, under the guidance of elastographic imaging, EUS-FNAB or EBUS-FNAB can improve the positive rate of diagnosis and avoid false-positive results.

**Table 2:** The diagnostic performance of EUS or EBUS elastography in differentiating benign and malignant LNs.

Study	Method	Study description	LN	SE (%)	SP (%)	PPV (%)	NPV (%)	Accuracy	Gold Standard
Knabe, *Surg Endosc* (46)	EUS	Qualitative (3 patterns)	40	100	64.1	75	–	–	Cytology, histology
Izumo, *Jpn J Clin Oncol* (48)	EBUS	Qualitative (3 patterns)	75	100	92.3	94.6	100	96.7	Histology
Korrungruang P, *Respirology* (49)	EBUS	Qualitative (3 patterns)	120	100	66.7	92.3	100	83	Histology, surgical pathology
Ching-Kai Lin, *Journal of the Formosan Medical Association (* 50)	EBUS	Qualitative (3 patterns)	206	64.7	85.6	71.6	81.3	78.2	Histology, surgical pathology
Fournier C, *Bronchology Interv Pulmonol *(51)	EBUS	Qualitative (3 patterns)	217	87	68	80	77	80.7	Histology
He, *Journal of Central South University Medical Sciences* (52)	EBUS	Qualitative (4 patterns)	68	85.7	76.9	85.7	76.9	82.3	Cytology, histology, surgical pathology
Ahmed Youssef Altonbary, *Diagn Ther Endosc* (53)	EUS	Qualitative (4 patterns)	40	87.5	41.7	83.3	50	60	Cytology, histology
Giovannini, *WJG* (47)	EUS	Qualitative (5 patterns), multicenter	101	91.8	82.5	88.8	86.8	88.1	Cytology, histology
Xu, *Gastrointestinal Endoscopy* (54)	EUS	Meta-analysis	431	88	85	–	–	94.6	Cytology, surgical pathology
Korrungruang P, *Respirology* (49)	EBUS	Quantitative (SR ≥ 2.5)	120	100	70.8	93.2	100	85	Cytology, histology, surgical pathology
Hussein, *Arab Journal of Gastroenterology* (55)	EUS	Quantitative (SR ≥ 4.61)	126	89.8	83.3	82.5	90.2	–	Cytology, histology, surgical pathology
Ahmed Youssef Altonbary, *Diagn Ther Endosc* (53)	EUS	Quantitative (SR ≥ 6.7)	40	99.9	57.1	99.9	64	77.5	Cytology, histology

EUS, endoscopic ultrasound; EBUS, endobronchial ultrasound; LNs, lymph nodes; SE, sensitivity; SP, specificity; PPV, positive predictive value; NPV, negative predictive value; SR, strain ratio.

With qualitative analysis of elastographic histogram, elasticity pattern and SR have been studied to evaluate the stiffness of LNs. Giovannini et al. firstly evaluated the ability of EUS elastography to differentiate benign from malignant LNs with elasticity pattern in 2006 (56). In this color-coded scale of elastographic patterns, yellow means normal tissue, green fibrosis, red fat, and blue malignant tissue. They conducted a multicenter study in 2009 and found improved specificity of 82.5% compared with 50% in the previous study (47). What is more, the sensitivity, specificity, PPV, NPV, and global accuracy of EUS elastography were 91.8%, 82.5%, 88.8%, 86.8%, and 88.1%, respectively, which were significantly better than the respective parameters of B-mode (56). In a study including 40 patients with a 4-point elasticity score, using the criteria of elasticity scores 1 and 2 as benign and elasticity scores 3 and 4 as malignant, the sensitivity, specificity, PPV, NPV, and diagnostic accuracy were 87.5%, 41.7%, 83.3%, 50%, and 60%, respectively (53).

Besides, some researchers qualitatively classified EBUS elastograms into three patterns (48, 50, 51): pattern 1, predominantly non-blue (green, yellow, and red); pattern 2, partly blue and non-blue (green, yellow, and red); and pattern 3, predominantly blue. With the use of the criteria of pattern 1 elastogram as benign and pattern 3 as malignant for differentiating malignant and benign mediastinal LNs with EBUS elastography, the sensitivity, specificity, PPV, NPV, and diagnostic accuracy were 90.6%, 82.6%, 71.6%, 94.7%, and 85.2%, respectively. But the central necrosis within malignant LNs and the fibrotic component within benign LNs may influence the accuracy of elastographic evaluations. What is more, the definitions of elastography patterns were subjective and may be hard to repeat by other researchers.

**Figure 8:**
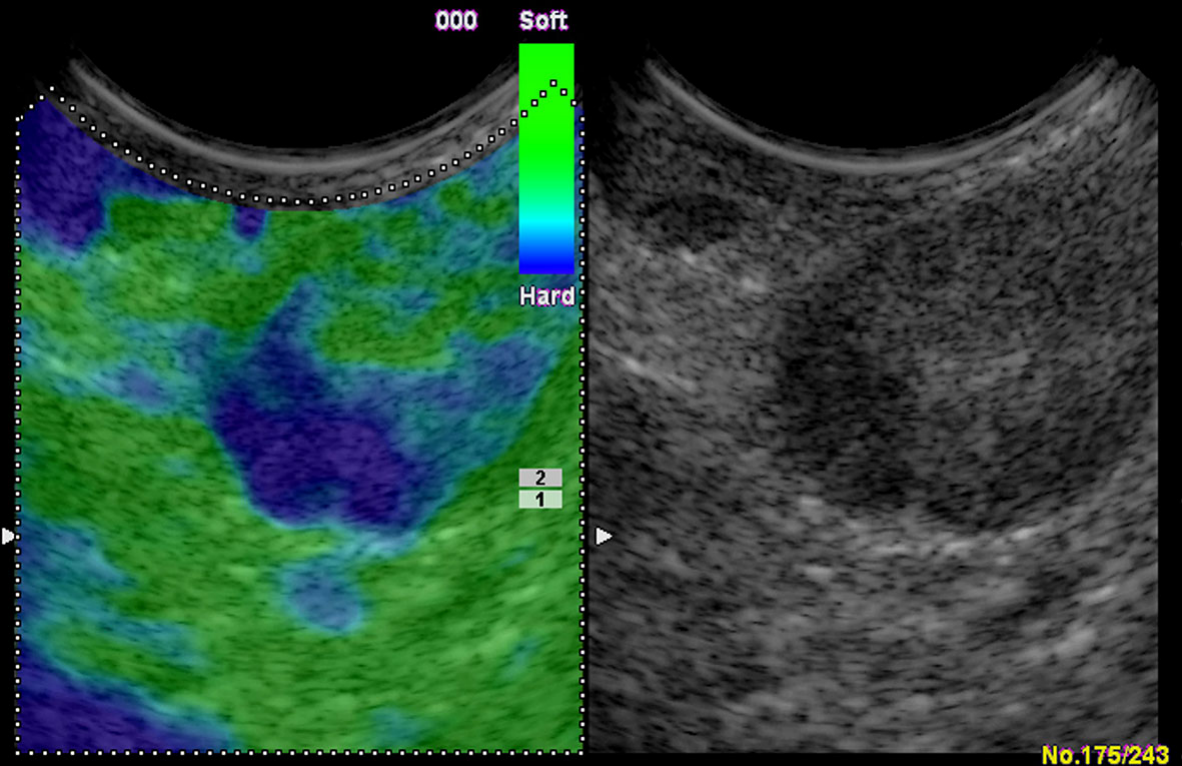
Colorectal carcinoma with presacral circumscribed lymph node metastasis in endoscopic ultrasound. The strain elastography reveals a typically harder (blue) area in the lymph node.

When judging malignant LNs with SR, previous research showed that with the cutoff point of SR >2.5, EUS elastography can differentiate benign from atypical malignant mediastinal LN sensitively (56). Okasha et al. reported that there were 89.8% sensitivity and 83.3% specificity in differentiating malignant LNs from benign ones with endoscopic UE while using the SR cutoff value >4.61 (55). Altonbary et al. found that the sensitivity, specificity, PPV, NPV, and diagnostic accuracy for differentiating benign LNs from malignant LNs were 57.1%, 99.9%, 99.9%, 64%, and 77.5%, respectively, with the mean SR cutoff value >6.7 (53). These studies reported the SR was more accurate than conventional EUS or EBUS, and EUS elastography combined with other sonomorphologic features is a potentially useful prognostic index differentiating malignant from benign LNs (**Figure 8**). Besides, a meta-analysis found that the sensitivity and specificity of UE in differentiating benign and malignant LNs were 88% and 85%, respectively (54).

However, the SR was generally calculated by two selected target regions, which makes it hard to precisely represent the stiffness of the whole LN. Thus, some studies used software to semiquantitatively analyze the color distribution of LN elastogram. Nakajima and his colleagues analyzed 49 LNs with stiff area ratio; they found that the sensitivity and specificity were 81% and 85%, respectively, for predicting metastatic disease, using a cutoff value of 0.311 for stiff area ratios (57). Sun et al. used a software and transformed the elastographic image into gray scale, which varied form 0 (all red pixels) to 255 (all blue pixels). This method could calculate the mean gray value inside the target and reflect the stiffness of the targeted LN. They found that non-small cell lung cancer (NSCLC) showed a higher gray value than small cell lung cancer (SCLC) (201.33 versus 196.37) (58). Ma et al. found that the blue color proportion (BCP) of LNs containing benign diseases was higher than that of normal LNs containing lymphatic tissue (33.3%, 49.0%, and 42.9% *versus* 27.0%), which revealed that the LN stiffness would increase in some diseases with a higher density of cells and vessels, like granulomas and pulmonary infection. These LNs might show the features of metastatic LNs if assessed solely by EUS elastography. However, the BCP in malignant LNs was remarkably higher than benign LNs (p < 0.001, 57.1% versus 31.1%). The highest average BCP was shown in lung squamous cell carcinoma (71.6%) (59).

According to published studies on the qualitative EBUS elastography in differentiating benign from malignant LNs, Korrungruang et al. found that two methods had similar diagnostic performances (49), but Lin et al. considered that qualitative EBUS elastography may be more suitable for clinical practice (50).

In the future, more studies should be conducted to compare the qualitative and quantitative EUS elastography in differentiating benign from malignant LNs, in order to find a more suitable, accurate method for clinical practice.

## Conclusion

UE is a promising method for measuring tissue hardness and has been widely used in differentiating reactive LNs, lymphoma, metastatic LNs, and other lymphadenopathy. Besides, EUS and EBUS elastography are non-invasive techniques and have been proven to be able to provide complementary stiff information for conventional EUS imaging; the positive rate of diagnosis of EUS-FNAB or EBUS-FNAB can be improved under the guidance of elastographic imaging. There are some studies that used elastography in cervical, axillary, mediastinal, and peritoneal LNs, but further studies with unbiased large-scale samples in different sites are still required. Also, the direct comparison between qualitative and quantitative elastography and new solutions for current elastographic limitations should be pursued. The current consensus for LNs diagnosis is that no single parameter has sufficient diagnostic performance, and the combination of UE and traditional US technology is conducive to the differential diagnosis of LNs (**Table 3**). In conclusion, UE can aid in the differentiation of benign and malignant LNs and has immense potential clinical values.

**Table 3:** Criteria on lymph node characterization using different ultrasound modes.

Lymphadenopathy	Reactive lymph nodes	Malignant infiltration	Lymphoma	Tuberculous lymphadenitis
More (most) likely				
B-mode	Preserved architecture, aspect ratio > 2, uniform cortex	Eccentric hypoechoic cortical thickening, aspect ratio < 2, boundary ambiguity, tissue edema around	Destroyed architecture, focal or global hypoechoic cortical thickening, usually without echogenic hilum, approximate sphere, pseudocystic appearance	Similar with malignant infiltration
Color Doppler	Lymphatic vascular structure	Peripheral or mixed vascularity, vascular distortion	Rich vascularity	Peripheral or mixed vascularity
Vascular resistance	Lower, RI < 0.8, PI < 1.6	Higher, RI > 0.8, PI > 1.6	Intermediate RI and PI	RI < 0.8, PI < 1.6
Strain elastography	1–2 points in 4-point rating elastography scale, SR in diffuse infiltration < 1.7	SR in diffuse infiltration > 1.7	Patterns 1 and 2 in five pattern elastographic score, dynamic changes occur after treatment	No data
Shear wave-based elastography	No data, most often normal architecture	Shear wave velocity > 3.03 m/s	Shear wave velocity > 2.45 m/s	No data

RI, resistive index; PI, pulsatility index; SR, strain ratio.

## Author Contributions

Conception and design: A-JY, X-WC, and CD. Drafting of the article: BW, QG, and YY. Critical revision of the article for important intellectual content: BW, QG, and J-YW. All authors contributed to the article and approved the submitted version.

## Funding

This work was supported by the Science and Technology Department of Hunan Province under Grant No. 2020SK52705.

## Conflict of Interest

The authors declare that the research was conducted in the absence of any commercial or financial relationships that could be construed as a potential conflict of interest.

## Publisher’s Note

All claims expressed in this article are solely those of the authors and do not necessarily represent those of their affiliated organizations, or those of the publisher, the editors and the reviewers. Any product that may be evaluated in this article, or claim that may be made by its manufacturer, is not guaranteed or endorsed by the publisher.
